# Isolated hydatid cyst in the medial rectus muscle: unveiling a rare orbital occurrence

**DOI:** 10.1186/s12348-024-00436-8

**Published:** 2024-11-12

**Authors:** Alireza Attar, Behzad Khademi, Mohammad Hassan Jalalpour

**Affiliations:** Department of Ophthalmology, Poostchi Ophthalmology Research Center, School of Medicine, Shiraz University of Medical Sciences, Shiraz, Iran

## Abstract

**Background:**

Orbital hydatid disease, while rare, should be included in the differential diagnosis of unilateral proptosis, particularly in endemic areas. Accurate diagnosis and comprehensive management are essential for effective treatment and favorable long-term outcomes.

**Case Presentation:**

A 12-year-old boy presented with a one-month history of diplopia and left-sided proptosis. A CT scan revealed a cystic mass within the left medial rectus muscle. Surgical intervention confirmed the presence of a hydatid cyst following cyst rupture and irrigation with hypertonic saline. The patient underwent a two-month course of albendazole therapy. Initially, the patient experienced persistent exotropia and diplopia, but at the three-year follow-up, he exhibited no diplopia or proptosis and only mild residual exotropia.

**Conclusion:**

This case underscores the importance of considering orbital hydatid cysts in the differential diagnosis of unilateral proptosis in endemic regions. Heightened awareness, accurate diagnosis, and a tailored therapeutic approach, including surgical removal and antiparasitic treatment, are crucial for successful management and improved long-term outcomes.

## Precise

Isolated orbital hydatid cysts are rare. Accurate diagnosis, even with intentional intraoperative cyst rupture, irrigation with hypertonic saline, and antiparasitic treatment, is essential for effective management and favorable long-term outcomes without recurrence.

## Introduction

Hydatid disease, resulting from the infestation of larvae of the tapeworm Echinococcus granulosus, exhibits varying incidence rates across different geographical regions. This disease is particularly prevalent in underdeveloped countries and areas where sheep farming is common [1].Hydatidosis can affect nearly every organ or tissue in the body through portal or systemic circulation [2]. In humans, the liver and lungs are the most commonly affected sites, with the central nervous system involved in 2–3% of cases [3]. While hydatid cysts can form in any organ, orbital involvement is especially uncommon, representing less than 1% of all cases [4]. Despite its rarity, orbital hydatidosis is a crucial differential diagnosis for unilateral exophthalmos in patients from regions where echinococcosis is endemic [5]. This study highlights a rare case of a young man presenting with proptosis and diplopia persisting for one month. Diagnosis revealed an isolated hydatid cyst, initially undetected until pathological examination.

This report adhered to the ethical principles outlined in the Declaration of Helsinki.

### Case report

**Fig. 1:**
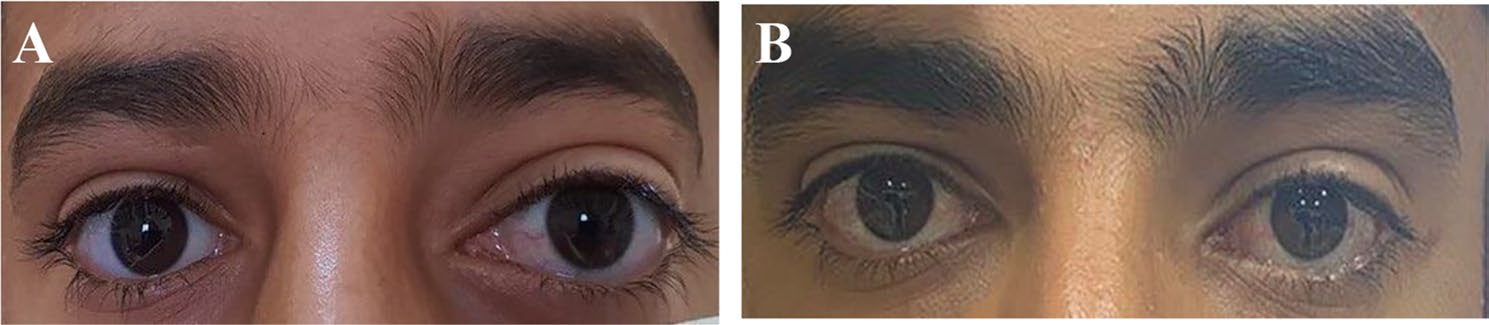
Face photo (**A**) Before Operation proptosis in the left eye, (**B**) Three years post-surgery

A 12-year-old male presented with complaints of diplopia and left-sided proptosis persisting for one month. He had no relevant medical history and no known exposure to animals such as dogs or sheep. His visual acuity was 20/20 in both eyes, with limited ocular motility in the medial direction on the left side (− 4). Hertel measurements were 15 mm on the right side and 20 mm on the left at a base of 92 mm (Fig. 1-A). The margin of the left optic disc was blurred. The remainder of the ocular examination was normal.

**Fig. 2:**
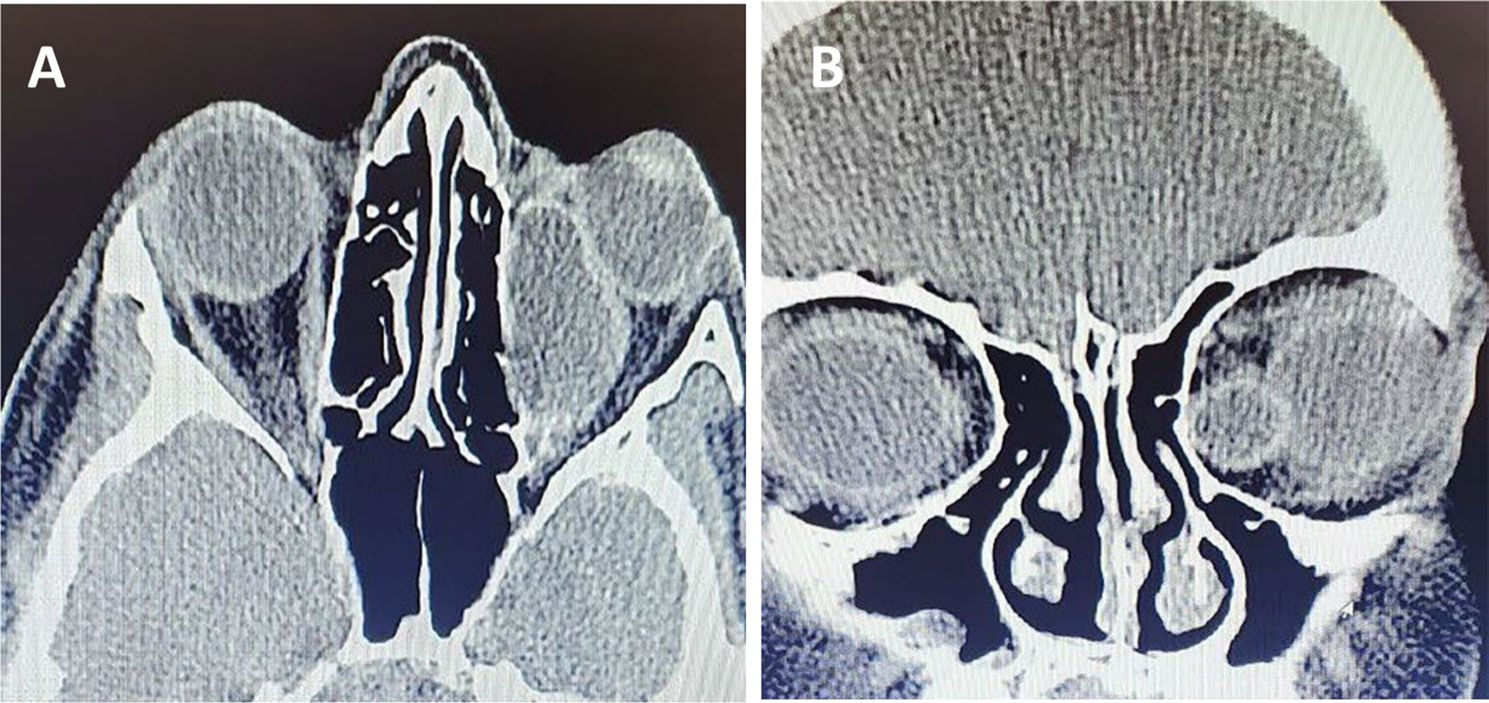
CT scans, axial (**A**) and coronal (**B**) orbital CT images revealed a circumscribed, well-defined cystic lesion in the medial rectus muscle

**Fig. 3:**
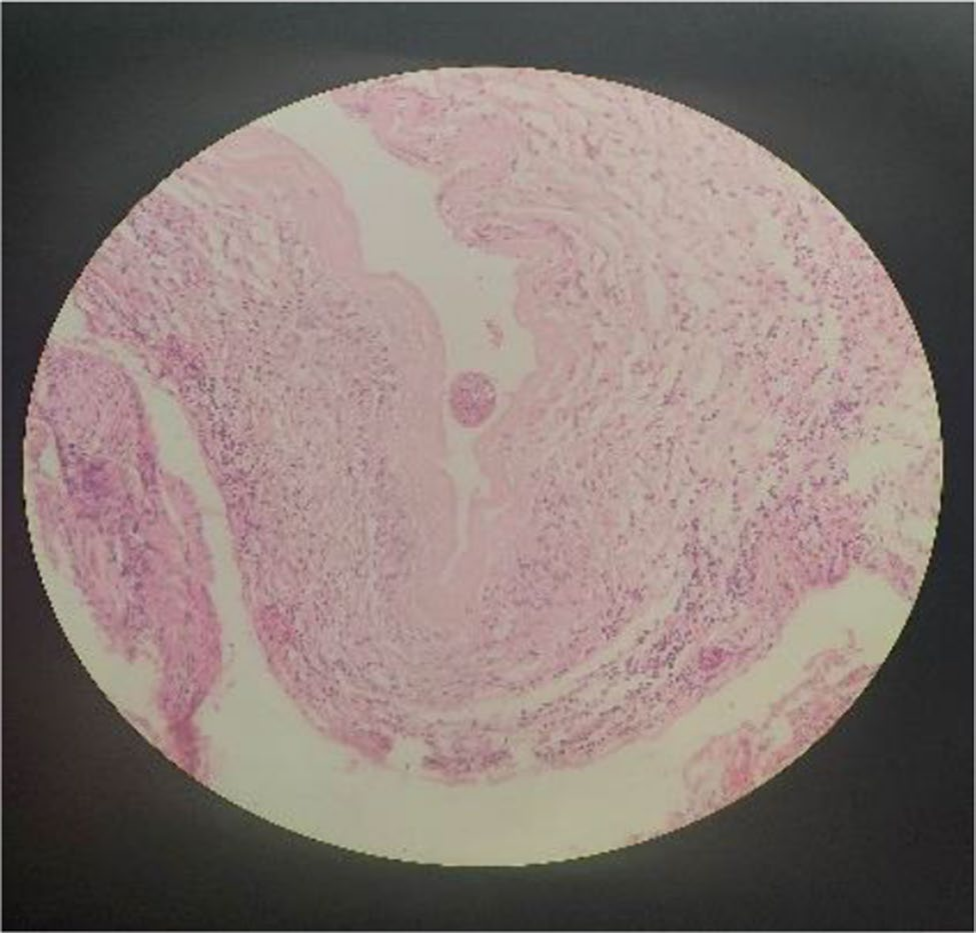
Hematoxylin and Eosin (H&E) staining; an acellular laminated membrane with germinal epithelium and daughter cysts containing protoscolices

A CT scan revealed a cystic mass within the left medial rectus. The eyeball was non-reducible and non-pulsatile. Axial and coronal orbital CT scans showed a well-defined, medio-inferiorly localized cystic lesion measuring 2 × 2 × 2.5 cm (Fig. 2). Routine blood tests, including erythrocyte sedimentation rate, yielded normal results. Blood chemistry analysis also revealed normal findings, without evidence of hypereosinophilia. In pathology, a sample stained with Hematoxylin and Eosin (H&E) revealed an acellular laminated membrane with a germinal epithelium and daughter cysts containing protoscolices (Fig. 3).

A safe approach for removing large intraconal masses, especially when there is concern about intentional rupture or en bloc mass removal, is to recommend lateral orbitotomy. However, the confined space in medial orbitotomy led us to perform a lateral orbitotomy with bone removal to obtain better access and space for dissection. Dissection was conducted laterally from the optic nerve and medially from the medial rectus muscle. During the operation, a large cyst was encountered. Due to the cyst’s considerable size and the risk of rupture during excision, complete removal was not feasible. To mitigate the risk of spreading the cyst contents, a controlled intentional rupture was performed at one point. This was followed by irrigation with hypertonic saline. The intentional rupture and subsequent irrigation were carried out prior to the excision of the cyst to ensure the cyst contents did not disseminate. Pathological examination confirmed the presence of a hydatid cyst. An abdominopelvic ultrasound, chest X-ray, and brain CT scan showed no additional hydatid cysts. The patient was started on albendazole (400 mg twice a day), which was administered for two months. However, after 10 months, his exotropia and diplopia persisted. He had a 30-prism diopter in primary position and limited motion in adduction of the left eye (− 2). A lateral rectus recession of 6.5 mm and medial rectus resection of 4.5 mm were performed.

Three years post-surgery, the patient had no diplopia or proptosis (Hertel measurements were 15 mm on the right side and 17 mm on the left at a base of 92 mm) and no recurrence of hydatid cysts, with only a 10-prism diopter in primary position exotropia remaining and limited ocular motility (− 1) in lateral gaze (Fig. 1-B).

## Discussion

Orbital hydatid disease, an exceptionally rare parasitic infection, is attributed to the tapeworm Echinococcus granulosus [6].Like many zoonotic diseases impacting humans, orbital hydatid disease typically arises from inadvertent ingestion of eggs expelled by definitive hosts, primarily canines [7]. The lungs, brain, spine, and orbit are more frequently affected in children and young adults compared to older individuals [3]. The orbit is a particularly rare site for hydatid disease, even in regions where the condition is common, accounting for less than 1% of all cases [4]. The clinical presentation of hydatid disease primarily stems from the mass effect exerted by the cyst on neighboring structures(6). This effect is particularly notable in regions with restricted space, such as the orbit [1]. In our case, consistent with typical presentations of hydatid disease, symptoms of an orbital hydatid cyst manifested gradually. Specifically, unilateral proptosis without associated pain was observed. Other reported symptoms in previous studies encompass unilateral proptosis with or without pain, visual impairment, periorbital pain, chemosis, and headache [8]. Accurate preoperative diagnosis and detailed localization are crucial for the effective management of orbital hydatid disease. Therefore, differential diagnosis should include consideration of other cystic mass lesions such as abscesses, cysticercosis, orbital hemorrhagic cyst, intraorbital conjunctival cyst and venolymphatic malformations [9]. Although an orbital hydatid cyst was at the top of the list of differential diagnosis, we were uncertain due to absence of daughter cysts on imaging. However, given that iatrogenic rupture of cyst could lead to anaphylaxis, as reported in previous cases, we decided to do lateral orbitotomy. Under controlled conditions, we intentionally ruptured the cyst to prevent the leakage of antigenic material. The pathology report subsequently confirmed the diagnosis of a hydatid cyst. In our case, orbital hydatid cysts manifested as unilocular hypodense cysts on CT imaging. However, it’s noteworthy that atypical hyperdense hydatid cysts can mimic other soft tissue orbital tumors, as observed by Sperryn et al. [10]. On CT, typical features of orbital hydatid cysts include a unilocular, non-enhancing homogeneous cyst with low density, akin to the visualization of the vitreous body [11]. If there is suspicion of a hydatid cyst, MRI is preferable for the differential diagnosis of soft tissue lesions. Orbital hydatid cysts are almost invariably situated in the superolateral and superomedial angles of the orbit, lying in or close to the muscle cone [12]. However, in our case, the hydatid cyst was located in the medial rectus muscle and presented with restrictive exotropia. This is the second reported case of intramuscular hydatid disease in the orbit [2]. Surgery is the most effective treatment for an orbital hydatid cyst. While medical therapy can be considered, it is generally less effective in intraorbital echinococcosis compared to other locations of the disease. In our case, both surgery and medical therapy were used and proved to be effective.

For surgical treatment, Radical surgery of the cyst is one option. Another option involves intraoperative aspiration of the cysts, which has the advantages of aiding diagnosis, reducing the size of the cyst, and causing the inner germinative layer to collapse, allowing the outer fibrous wall to be safely tented and snipped open [13].In the event of intraoperative cyst rupture, irrigation with hypertonic saline and hydrogen peroxide is recommended to neutralize daughter cysts and mitigate the risk of further dissemination [14]. In our case, due to the large size of the cyst, intentional rupture followed by irrigation with hypertonic saline was deemed necessary.

After confirmation of the hydatid cyst, we initiated treatment with albendazole. Albendazole is potentially more suitable for treating orbital cysts, as it has a broad spectrum of antiparasitic activity [15]. In contrast, medical therapy with mebendazole is likely ineffective in orbital hydatid disease and has no known therapeutic effect on hydatid disease of the central nervous system. Mebendazole crosses the blood-brain barrier poorly and may also penetrate the orbital region insufficiently [16].

In conclusion, although orbital hydatid cysts are rare, they should be considered in differential diagnoses in endemic regions. Accurate diagnosis, combined with intraoperative cyst rupture, irrigation with hypertonic saline, and antiparasitic treatment, is essential for effective management and favorable long-term outcomes without recurrence.

## Data Availability

No datasets were generated or analysed during the current study.
